# Suicide and Self-Harming Among Young Women: A Qualitative Exploratory Study in Southern Punjab, Pakistan

**DOI:** 10.3390/healthcare13111284

**Published:** 2025-05-29

**Authors:** Farooq Ahmed, Eileen Yuk Ha Tsang, Razia Anjum, Najma Iqbal Malik, Sidra Zia, Rashed Nawaz, Jeffrey S. Wilkinson, Yueyao Fang

**Affiliations:** 1 Department of Anthropology, The Islamia University of Bahawalpur, Bahawalpur 63100, Pakistan; sidrazia2010@hotmail.com; 2 Department of Social and Behavioral Sciences, City University of Hong Kong, Hong Kong 852, China; yueyafang2-c@my.cityu.edu.hk; 3 Department of Psychology, Bath Spa University, Ras al Khaimah 71705, United Arab Emirates; razia@bathspa.ae; 4 Department of Psychology, University of Sargodha, Sargodha 33600, Pakistan; najma.iqbal@uos.edu.pk; 5 School of Public Health and Health Nutrition, Luohe Medical College, No.148, Daxue Road, Yuanhui District, Luohe 462002, China; rashednawazpk@gmail.com; 6 School of Journalism and Graphic Communication, Florida A&M University, Tallahassee, FL 32311, USA; jeffrey.wilkinson@famu.edu

**Keywords:** suicidality, rural women, marriage, Paraphenylenediamine (PPD), Southern Pakistan, thematic analysis

## Abstract

Background: Suicide and self-injury are serious public health concerns, especially in young populations, owing to multiple social, cultural, and gender determinants. Qualitative evidence exploring narratives regarding the factors behind suicide among young women is rare in Pakistan. Objective: The present study aims to explore the complex dimensions of suicide or self-injury among young women of Southern Punjab. Methods: Semi-structured interviews were conducted in a marginalized district in South Punjab, with participants consenting to in-person meetings at their homes or phone interviews. We collected detailed accounts of fifteen deceased girls or self-harm survivors, with insights provided by close relatives of the victims. Results: Our findings identified several conducive factors to suicidality, including receiving insults in front of others, low self-esteem, household pressures, work burdens, unfulfilled romantic desires, feelings of worthlessness, cheating in love, marriage without choice, and engagement in risky behaviors. These causes could be categorized into personal (such as an inferiority complex), social (a lack of family support and frequent conflicts), and cultural factors (forced marriages). Conclusions: Our study advocates for empowering women through education and restricting access to suicide means, such as pesticides or Paraphenylenediamine (PPD). Moreover, the government should take strict measures to discourage the forced marriage of young females in rural contexts. This study highlights the importance of integrating suicide prevention initiatives with research efforts within Pakistan’s healthcare system.

## 1. Introduction

Suicide is a major global health issue, particularly among individuals aged 15–30, with over 800,000 deaths annually [1,2]. It is a leading cause of mortality in several low- and middle-income countries (LMICs), with nearly 45% of global suicides occurring in just two countries: China and India. In China, the suicide rate is notably higher in rural areas compared to urban regions.

The accurate reporting of suicides to the police, health systems, and the WHO is often hindered by the potential legal and social repercussions faced by the victims’ families. Official mortality data on suicide at the national level in Pakistan are unreliable because family members often attribute suicide to “accidental” deaths or “natural death”. However, the WHO’s estimates show a suicide rate of 7.5 per 100,000 individuals, a 2.6% rise since 2000, with approximately 270,000 annual cases of deliberate self-harm (DSH) [1]. The WHO prioritizes the elimination of suicidal acts through initiatives such as the Mental Health Gap Action Program (MHGAP), and preventing suicide is included in its sustainable development goals [3].

Suicide is defined as a fatal self-inflicted act with clear evidence of an intent to die [4]. Around 80% of all suicides occur in LMICs [5]. In 2015, the global suicide rate was 10.7 per 100,000 people, equating to approximately one death every 20 s. Suicide represents 1.4% of all deaths and ranks as the 15th leading cause of death worldwide [3,5]. In LMICs, the suicide rate among women is very high [6]. Evidence indicates that women’s suicidality is higher in Asian countries such as China, India, Pakistan, and Nepal [7].

In general, suicidal behavior is correlated with conflict, tragedy, violence, abuse, loss, and a sense of isolation [1]. Both self-harm and suicide are prohibited and considered illegal under Islam and the law of the country. Self-harm or suicide is viewed as a sin in Islam. In Islam, suicide is deemed an illegitimate act, and those who commit suicide are believed to be denied entry into paradise [8]. Legally, suicide is a violation and is classified as a violent crime. For a suicide attempt, Section 325 of the Pakistan Penal Code (PPC) mandates penalties of up to one year’s imprisonment, fines, or both [9]. As per law, suspected suicide or self-harm cases ought to be reported to selected medicolegal centers (MLCs). However, to avoid legal repercussions, family members usually request initial treatment at private hospitals, often discharging themselves against medical advice due to the fear of prosecution, financial limitations, and societal shame. Suicidal behavior is under-reported due to these challenges in Pakistan.

The World Health Organization (WHO) aims to enhance the public understanding of the health impacts of suicide and self-harm while also assisting countries in developing and strengthening comprehensive, multisectoral strategies for suicide prevention within a public health framework [3]. Suicide often goes unrecognized or unreported due to its sensitive nature and the ongoing taboo surrounding it [10]. The risk of suicide is linked to traumatic events and environmental changes [11,12].

Lester’s work, *Why Women Kill Themselves*, is a comprehensive, cross-disciplinary collection that includes several insightful sections on the psychological mechanisms associated with suicide and the spiritual themes found in women’s suicide notes [13]. However, the collection has its shortcomings as it highlights a prevalent tendency among researchers to examine women’s suicide with their biology, particularly their reproductive cycles. It is noteworthy that between 1999 and 2012, the increase in the suicide rate among women in Northern Ireland was slightly higher than that among men [14], yet their deaths received minimal attention. For example, suicide in women is rarely covered in the media, and even more concerning is that women often go unrecognized in suicide prevention policies [15]. Research has explored how social factors, life events, and aspects of female identity may be linked to the deaths of women who died by suicide [16].

Only few previous qualitative studies showed that an early age of marriage, the lack of independence in the choice of the male partner (arranged marriage customs), pressure to have children early in the marriage, the desire for a male offspring, economic dependence on husband, a joint or extended family system, and domestic violence are critical sociocultural factors that determine women’s health. There is little evidence that shows that gender is an important determinant of suicide and self-harm in Pakistan, especially when considered in the context of marital status among young rural women. There is a dearth of qualitative studies that focus on social and cultural determinants of women’s suicide; therefore, the present study explores the causes of suicide and self-injury among young rural women from South Punjab.

## 2. Methodology

### 2.1. Background and Study Setting

In this study, we investigate suicide among young rural women of Southern Punjab regions, examining reports of suicide and self-harm, their current mental health status, and their future intentions regarding suicide. Pakistan, a country with a population of 220 million, comprises four provinces (Punjab, Sindh, Baluchistan, and Khyber Pakhtunkhwa) with various languages and cultural norms. Southern parts of the country are marginalized, including the Southern Punjab districts in Punjab province. South Punjab is a highly disadvantaged region that lacks adequate hospitals, psychiatrists, and clinicians. In southern districts, young people are deprived of essential services such as healthcare, education, safe drinking water, food, and sanitation, and children suffer from diarrhea and malnutrition. Rural areas in South Punjab are marginalized with limited health facilities, particularly for women, who face significant barriers to accessing healthcare, resulting in missed opportunities for better health [6,17]. Contraception prevalence is low, and women have to experience high fertility and low birth spacing. Poverty is widespread, and communities often face moderate to severe water and food insecurity experiences [18]. Household male members have to migrate to Gulf countries as manual labor for their survival and subsistence, while women have to work as domestic household servants [19,20]. Many poor females suffer from domestic and gender-based violence and insulting behaviors from their in-laws after marriage due to their unemployment, poverty, and low education level [21,22,23].

### 2.2. Participants

#### 2.2.1. Sampling and Inclusion/Exclusion Criteria

This qualitative study is based on a purposive selection of victims’ relatives, siblings, neighbors, and teachers from the South Punjab region of Pakistan. The respondents in this research were identified through close connections, who were engaged to explore the circumstances that led them to believe there is no way out. We also discovered what methods and means they used to harm themselves and the impact on their health.

This study specifically included participants who recently had lost a close young female sibling, relative, or friend. Only young and rural women between 18 and 25 from the most marginalized districts of South Punjab, who have committed or survived suicide attempts, were eligible to be included. In total, fifteen respondents showed willingness to participate in this study and provided their verbal consent. In the final round, nearly thirteen cases of young women between the ages of 18 and 25 years could be narrated. Most of our study involved cases of victims who died after suicide attempts, while a few cases were those who self-injured.

#### 2.2.2. Interviews and Data Collection Procedure

The data were collected through semi-structured interviews and analyzed through probing, exploratory scrutiny, fact-finding, and a comprehensive examination of the circumstances in which individuals feel trapped. We selected interview locations that made respondents feel safe and comfortable. The use of audio recorders was with their permission. Some participants did not allow recording; therefore, we took notes respecting the participants’ traditional views and comfort levels. The interviews were conducted in a flexible format, lasting between one and two hours, and in the local (Seraiki) language. We fostered a warm and inviting atmosphere during the interviews and asked participants about the motivation for committing suicide or self-injury. They were asked to narrate the important and relevant parts of the victim’s life story. Participants were allowed to withdraw at any time without consequences. They were allowed to take a break if any difficult situation arose.

### 2.3. Thematic Analysis Procedure

**Figure 1:**
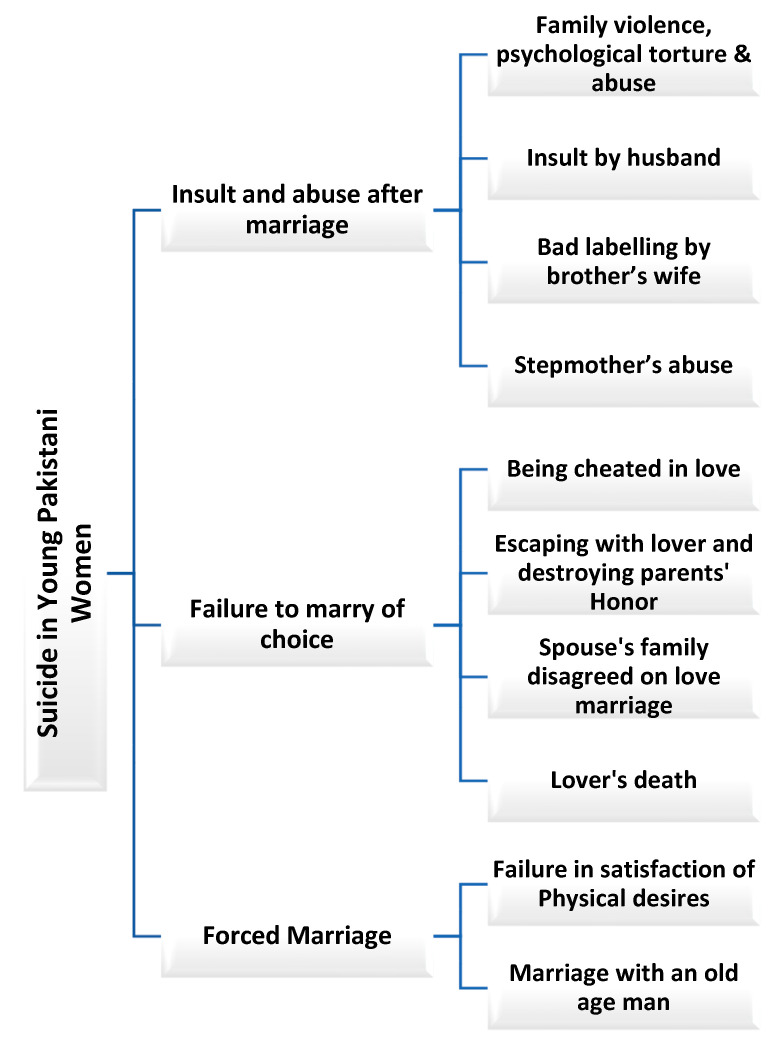
Suicidality among young women in Southern Pakistan.

Various steps were followed using Braun and Clarke’s framework for a thematic analysis procedure [24]. Soon after data collection from consenting participants using purposive sampling, all thirteen semi-structured interviews and field notes were translated from the local language to English. Three authors (FA, SZ, and RA) familiarized themselves with the data through the repeated reading of transcripts. Subsequently, in the next stage, recorded interviews were transcribed, and a coding frame was developed. Two authors (FA and SZ) used Nvivo for line-by-line coding to identify meaningful units. Then, two authors (FA and NIM) grouped several codes into themes and edited them through iterative discussion. Finally, researchers cross-checked themes against raw data and detached discrepancies via consensus. Themes were organized based on the analyzed data using a qualitative interpretative approach. Using this systematic approach, we ensured rigor and transparency in originating insights from respondents’ self-reported experiences. Thematic analysis provides a rigorous framework for in-depth qualitative exploration, allowing the systematic organization and interpretation of complex data. With participants’ informed consent, we collected and analyzed self-reported experiences through semi-structured interviews, ensuring rich, detailed responses for evaluation. The themes and sub-themes are shown in the diagram (Figure 1).

### 2.4. Ethical Consideration

Department of Anthropology at the Islamia University of Bahawalpur granted ethical approval for this study (IUB/ANTH/99; date of approval 5 June 2024). Before participating in the study, all respondents were informed about its nature. Informed consent was taken from the respondents. Privacy, confidentiality, and anonymity were fully ensured. We did not disclose the real names of participants to protect their identities. Moreover, fictitious numbers were used to hide the original identity of the suicide and self-harm cases.

## 3. Results

Table 1 gives the sociodemographic characteristics of the study respondents. Most women were young, less educated, rural, and from a lower socioeconomic background. Many of them used poisonous substances to kill themselves, and marriage-related issues were behind the underlying determinants.

### 3.1. Theme One: Forced Marriages or Marriage Without the Choice of Young Girls

Women are forced to marry according to the will of their parents, family members, and relatives. However, these exchange marriages do not guarantee fulfilling their emotional and sexual needs. Multiple times, the age factor in these marriages is not considered. Exchange marriages without consent often stop sexual needs from being satisfied and cause suicidal ideation among rural females.

#### 3.1.1. Protest Against the Forced Marriage

Marriage with an aged person was the main cause, as one woman reported. Her old husband was unable to fulfill the sexual desire of her young wife. For this reason, the young bride was angry with her parents, but no one bothered to understand her feelings. On her first suicide attempt, some relatives arrived on time and carried her to the hospital, where her stomach was washed and her life was saved. After some time, she again attempted suicide but could not survive this time, and she passed away. One lady explained about her old uncle’s exchange marriage (locally called watta-satta).

*“My 70-year-old uncle got married by exchanging her son’s daughter with the groom’s brother. Once I asked, Are you happy with this old uncle? She said, ‘he can’t fulfill my desire. Wealth is not sufficient. Please ask my parents, “I can no longer live with him. Otherwise, I would take my life.” ‘Everyone was aware of her critical situation. One day, in the absence of my uncle, she attempted suicide, but one family member saved her life. She warned, ‘If you don’t divorce me, I will do it again.’ But no one could take it seriously. After some time, she took a black rock and ate it with some juice. This time she succeeded in dying”.*

Self-harm due to forced marriage is common. One respondent explained how a young girl in their family attempted suicide due to forced marriage:

*“She did not agree to marry an old man and attempted suicide. When we reached, she had drunk all the nail-polish remover, and her family members were crying. Her stomach was bulging inside her belly, she was not breathing well. She was catching her neck. My husband brought her to the hospital. The doctor told her that her breathing pipe would be disturbed for her whole life”.*

#### 3.1.2. Cultural Violence as the Motivating Determinant of Suicide

Suicide was committed as a form of protest against cultural violence, as remarked by a woman.

*“The future of women is decided without their consent, by the parents and family elders, and soon after marriages, domestic disputes and unsustainable relationships erupt with husbands, which forces women to commit suicide”.*

A mother of a 25-year-old female suicide victim reported the following:

*“My husband disliked that her daughter disobeyed his decision to marry. I failed to convince him, but he deemed his daughter’s confrontation an abuse of the local norms and customs. This controversial marriage made her depressed. My well-educated daughter decided to kill herself soon after she was forced to marry her uneducated cousin. My husband was ready to withdraw from his stance, so our daughter thought it better to lose her life”.*

The custom of honor killing is very relevant in the rural context of South Punjab. According to a mother, “kalakali” is the main issue for the increased suicide cases.

*“Kalakali means when a married woman is caught with her boyfriend, the family honor is considered ruined. Women in that case often prefer to die otherwise, they are sold out or murdered. The reasons to commit suicide are many, but the main causes are misbehavior, domestic issues, and violence by husbands and relatives. We did not report the suicide case to the police and kept it secret to evade social stigma”.*

### 3.2. Theme Two: Insulting Behaviors of Relatives and Suicide Among Women

#### 3.2.1. Violence, Stigma, and Depression

Several reasons were identified for suicide and self-harm: violence, stigmatization, mental torture and labeling, and the stepmother’s abuse and insults. Insulting behavior from family members and in-laws is the leading reason for suicide in young women. One respondent discussed the suicide of her aunt:

*“My aunt fell in love; her boyfriend sent his family to propose, but her parents refused, saying that since your elder sisters are still unmarried, how can you marry? After this, her family members used to abuse her verbally and physically. One day, she came in front of the train and was no more”.*

Embarrassed by her brother’s and his wife’s behavior, the 18-year-old girl heard that someone had died by suicide by eating kala pathar [PPD] with a glass of sweet juice. Abused by her brother’s wife, she also prepared her mind to do the same. She went to the shop, bought and ate PPD with juice, and died by suicide. Her relatives called her again and again, but she was missing. Her younger brother’s daughter went to the room where she was vomiting, but after some time, she was no more. Stigmatizing someone might be a risk factor.

One aunt talked about her niece’s sudden death at a young age in the following words:

*“My niece was young, energetic, and talented. Her brother’s wife was strict and cruel; she treated her like a maid. Her mother was a social lady and often used to leave her alone in the house with her brother’s wife. She was asked to wash clothes and utensils and cook food. Even after her work, her brother’s wife was not happy and used to label, stigmatize, and abuse her all the time. One day, she bought a packet of henna with black rock (PPD) and ate it with juice. After some time, her brother’s wife called her to make the loaf, but there was no answer. Her daughter went to check her in the room, but she was vomiting, and later she died”.*

Black rock is a hair-dyeing mixture with red henna. It is available in every local market, small shops, and even in food shops. Everyone can buy it very easily. It gives a very luminous color to hair, but it can kill within a few minutes if eaten.

The stepmother’s violence and labeling were reported as the reason for her taking her life. One female told the story of her niece:

*“My niece was a young, beautiful, and intelligent girl. But for some time, she was very disturbed and disappointed with her stepmother. She abused her in front of her dad, but her dad never interfered and supported. She came to me and was weeping and said, ‘Aunt (father’s sister), I will not go to my house because my stepmother pains me and labels me.’ The next day, we heard that she had eaten the black rock, which she reportedly brought for dyeing the hair. The main reason was consistent mistreatment of her stepmom with her and often labeling her as a bad character woman”.*

#### 3.2.2. Insult in Front of Other Family Members

Insult in front of family members strongly motivated suicide among women. One respondent who attempted suicide but was saved explained how her husband’s insult caused suicidality:

*“It was his habit to insult me before our children. One day, I told him, Now our children are older, please don’t insult me in front of them.” He replied, ‘I don’t care about you and your children, leave me, I will never die without you.’ I was shocked and went to the room, took the pesticide, and drank it. But later, I was saved by the doctors”.*

Women self-harmed when their self-respect was crushed. One lady elaborates as follows:

*“My husband was bad-tempered and used to insult me in front of my family members. He often put me in a painful situation. Once, I saw a spray bottle of organophosphorus compounds (pesticides) in anger and drank a few sips. But my family members managed to take me to the hospital, and I was saved”.*

### 3.3. Theme Three: Failure in Marriage or Love Due to Unfaithfulness

#### 3.3.1. Failing to Marry Due to Cheating

Failing to marry due to cheating is another major reason for suicide among women. Marriage reportedly failed owing to a range of factors, including cheating, refusing to marry the boyfriend, destroying the parents’ reputation (honor), resistance from the spouse’s family about a love marriage, and the boyfriend’s death.

Quarrelling with a girlfriend was also highlighted as one of the potential causes. One lady elaborates on her sister’s suicide case due to infidelity:

*“My sister loved a boy, but when she insisted on marrying her, he shouted and said No, I have many girlfriends, and I can’t marry all of them. You are not the only one with whom I talk. If you ask me to marry you, I will block your number from my contact list. And he literally did this. My sister was very sensitive; she could not accept this insult and hanged herself. After a few days, the police arrested him, and he agreed that they both quarreled”.*

Women commit suicide when they get cheated on and their family’s reputation (honor) is spoiled. One girl elaborates on her classmate’s suicide:

*“My classmate from a very ethnically strict family fell in love. People told her not to meet with that guy. He belonged to a very rich family, maybe he will cheat you. She said, ‘Love is blind. I cannot live without him. I know my parents are strict, but this love is not in my control, it’s in my heart.’ Once she escaped with that guy, but unfortunately, the boy left her. After some days, my classmate came to her parents’ house, but they did not want to accept her. They said, ‘You buried our honor, we wish you were buried under this earth.’ She did not accept this treatment from her own parents and committed suicide with the black rock”.*

#### 3.3.2. Failure of a Love Marriage

Failure of a love marriage can also trigger suicide among sensitive females. A girl narrated a story about her sister:

*“She was young, beautiful, with long hair, but was looking sad. I tried to investigate what happened. She revealed: ‘I think I will die soon because I often dream, I am dying. One night I dreamed that I died and there was a moringa tree upon my grave.’ I said to her, Don’t feel stressed, just donate some slaughter. It was just a dream. My sister fell in love with my cousin, but the boy’s mom did not agree. One night, my sister used insecticide crushed tablets, which were used for the protection of wheat. In the early morning, she fainted and all family members wanted to save her life, but it was too late”.*

Marriage outside of the family is disliked. Many girls commit suicide when their families do not support their marriage. The death of a lover was mentioned as a strong reason for suicide. A teacher narrated the story of her student:

*“One of my female students fell in love with someone, and the boy was from another caste. She discussed this matter with her brothers, who got angry when she discussed this matter with them. They said, Go and marry, but we will give no piece of land to you. We will not allow them to become rebels like you. After some period, that girl came to me and told me, ‘My love has died due to a medical issue, my brothers also don’t accept me, now I have no reason to live, I want to die. After that, she hanged herself and died”.*

## 4. Discussion

In this study, we identified the causes of suicide among young rural females of South Punjab, Pakistan. The primary factors contributing to suicide include poverty, insufficient family support, violence, insult, fear, conflicts within marital life, and forced marriages. According to Agnew’s General Strain Theory (GST) [25], there are three primary functions: the blocking of personal goals, the absence of positive stimuli, and the presence of negative stimuli. The first type of strain involves the obstruction of personal goals. The second type of strain refers to the loss of a loved one, such as a partner or significant other. The third type of strain encompasses traumatic events like inhumane treatment, neglect, and abusive behavior [25].

### 4.1. Socioeconomic and Demographic Factors

Understanding the social dynamics surrounding suicide is crucial, as it highlights the importance of addressing and reducing suicide risks to implement more effective early interventions [26]. Evidence strongly supports that poor economic status and deprivation are significant risk factors for suicide [27,28,29]. A recent systematic review of contributing factors revealed that two major reasons behind suicide are unemployment and low socioeconomic status [30]. The study argued that suicide decisions are not always illogical, as according to the classic economic theory of suicide, suicide is rationalized after analyzing the costs and benefits. The rise and fall of suicide rates vary with unemployment, the level of economic development, and educational achievement. When adolescents and young people face adversity and challenges at a young age, it becomes a significant risk factor for suicide. Similarly to our study, a recent study also highlights that high suicide risk was associated with low educational attainment and ethnic disparities [31].

Our findings are perfectly corroborated by a recent qualitative study from neighboring India that showed that “interpersonal stressors, particularly in young age females”, shortly before a suicide attempt produce distorted cognitions and irresistible emotions of anxiety or anger due to relationship issues with partners or family members deeply rooted in sociocultural norms. This study recommends fostering life skills for young people and high-risk groups, such as women, curbing access to harmful substances, and involving family stakeholders [31,32].

Factors within the household, such as strained relationships and emotional neglect, contribute to feelings of hopelessness and despair [33,34,35]. When desperation, dissatisfaction, and abuse intersect, they significantly increase the risk of suicidal behavior [36,37,38,39]. In Pakistan, mental health awareness remains limited, leading many to attribute psychological distress to supernatural reasons such as divine punishment, curses, or magic [40]. the literature has revealed that individuals who engage in self-harm or attempt suicide often experience higher levels of depression [41,42]. Consequently, vulnerable members resort to sacrificing their own lives or those of their children, or offer symbolic items like roosters, goats, or prayers in the expectation of alleviating their suffering.

### 4.2. Gendered Vulnerability and Violence

The findings reveal that women often face harsh criticism from their family and relatives, which leads to feelings of isolation and hopelessness. Research suggests that gender-based vulnerabilities may contribute to an increased risk of suicidal behavior. Insults in front of family members and the mistreatment of women are significantly linked to suicidal behavior. Rural women, in particular, are disproportionately affected by violence, discrimination, and emotional distress, which have been linked to suicidal actions [43]. The literature showed that corporal punishment, spiritual abuse, collaborative brutality, living in oppression, and being imprisoned in a household caused suicide [44]. Evidence indicated that family violence and social humiliation produced emotions in which no other choice is recognized except suicide in India [45] and Nepal [46].

Research indicates that violence poses significant risks to women’s mental health. In tribal Pashtun culture, parents often arrange marriages for their daughters and sisters without considering their preferences. This lack of agency can lead to household conflicts and disputes with husbands, which, in turn, may result in women attempting self-harm [47,48]. A study found that ongoing and severe mistreatment from partners has detrimental effects on women’s psychological well-being [49,50]. Some cases also reported injuries resulting from violence against women [24]. Increasing evidence of physical, sexual, and emotional abuse indicates a decline in women’s mental health [51,52]. Similar research suggests that violence is more prevalent in LMICs where resources are distributed unequally [53].

### 4.3. Marital and Family Dynamics

Women identified marital conflicts as a major reason for suicide [54]. A study indicated that marital conflict, abusive arguments, and physical violence against women contribute to suicide [41]. Suicides among women in the Malakand division were prompted by marital issues, violence, and other factors [55]. Evidence shows that suicidal behavior among adolescents and young adults, caused by an unfriendly family atmosphere, broken interactions between family members, and an absence of perceived family support, might lead to suicide. Therefore, these causes must be anticipated before suicide prevention planning [56]. A study in Pakistan [57] found that adverse childhood experiences led to significant stress, anxiety, and insecurity among children. A study found that if a woman experiences stigmatization, violence, labeling, and abuse from her family, there is a critical need for social support programs such as shelters, group homes, and social networks to assist [35]. In Uganda, women often resort to suicide in response to household problems and a lack of social support [58].

Additionally, forced marriage is considered a violation of women’s and girls’ rights. Partners are often pressured and coerced into marriage through domestic violence [59]. This coercion undermines their freedom and dignity, effectively forcing them into the role of a spouse. Studies have shown that suicidal behavior among adolescents and young adults is influenced by perceived domestic factors, including aggressive home environments, critical comments, a lack of validation from mothers, and insufficient emotional support from family members [56]. Parental conflicts disrupt the home environment. Parents who are unwilling to engage in positive conversations, parental separation, and abuse contribute to suicidal behavior [56]. Research has identified that issues like a lack of respect and empathy, along with spiritual neglect from the family, are primary reasons for self-harm [60]. Another study conducted in Nepal [54] found that poor marital conditions, abuse, and neglect by husbands often contribute to suicide among women.

Our results indicated that some women died by suicide because of unfulfilled sexual needs as they were forced to marry aged men. A study conducted in Sweden highlights that in heterosexual relationships, men and women are on an equal footing when it comes to the key aspect of “sexual pleasure on equal terms”. They achieve mutual sexual desire and satisfy each other’s needs [61]. In Pakistan, it is rare for individuals to choose their spouses [62]. Pridmore [63] notes that major causes of suicide include issues related to fate, well-being, freedom, and honor. Researchers have observed similar issues in the district of Chitral, Khyber Pakhtunkhwa, Pakistan, where conflicts, unmet desires, and a lack of hope are prevalent [64].

### 4.4. Family Honor and Ethnic and Cultural Violence

This study reveals that the respect and dignity (izzat) associated with family or caste are significant causes of suicide in rural and tribal areas. People are extremely concerned about the honor of their family (Baradari). Daughters have little opportunity for love marriages, and if a girl falls in love, her parents may respond with anger, violence, and aggression. If a girl marries and runs away with a boy, her parents often refuse to accept her back, closing their doors to her rebellious actions. Without support from their parents and with limited interaction with family, many women view death as the only solution to their problems. In Nepal, the shame and social exclusion experienced by girls who marry and leave school at a young age are largely responsible for their disallowed attachment connections [54].

Feelings of anger, depression, irritation, and dissatisfaction become sources of strain, potentially leading to crime or suicide [65]. In South India, cultural values, normative behaviors, and family structures prevent clinicians and therapists from referring suicide attempt survivors for further help [66]. Evidence from other South Asian countries, such as Nepal [67], also suggests that a family’s izzat (social status) is a major concern. Hagaman [68], a medical anthropologist, suggested adding familial perspectives and experiences to high-level policy and planning.

### 4.5. Methods and Means of Suicide

Research indicates that victims utilized various methods for suicide, including black rock (often mixed with juice), insecticides, pesticides, hanging, and nail polish remover. While all these methods pose significant risks, black rock was identified as the most lethal, leaving no chance of survival. Many women used “black rock”, a substance commonly found in grocery stores that is typically used for dyeing hair. According to previous studies, women have been known to use benzodiazepines or organophosphate pesticides as methods of suicide [69]. Given the severe dangers associated with pesticides, some studies [70,71] suggest that rural and agricultural communities should take measures to restrict young women’s access to these substances.

### 4.6. Limitations and Strengths of the Study

Our study has some limitations. First, it is specific to the context of the Southern Punjab province of Pakistan, which may limit its generalizability to other ethnic or cultural settings of the country. Secondly, the sample size is not very large, which could further restrict the broader applicability of the findings. Thirdly, interviewing relatives may also introduce potential exaggeration or personal bias in our qualitative data. Despite these limitations, there are key strengths of this study. It is a novel contribution to exploring young and rural women and sociocultural and marital factors, a topic that has rarely been examined before. Furthermore, the use of in-depth interviews and rapport-building in an ethnographic approach adds depth and credibility to this research.

## 5. Conclusions

In this study, we investigated the primary motives behind women’s suicides in the South Punjab region of Pakistan. Our findings revealed that the prominent factors revolved around marital life, including relationship failures, desperation, violence, family torture, abuse, impatience, poverty, forced marriages, and fear of their husbands. Forced [or exchange] marriages must be closely monitored, and those involved should be held legally accountable. Empowering women and ensuring their economic independence can eliminate their dependency on in-laws and relatives, thereby preventing the insult and abuse often faced by dependent women. Providing emotional health counseling for young women can offer crucial support and improve their quality of life. Additionally, there is a pressing need for improved emergency care, and the government should take steps to ban the sale of harmful and deadly products. This highlights the importance of Integrating suicide prevention initiatives with research efforts within Pakistan’s healthcare system.

## Figures and Tables

**Table 1:** Sociodemographic characteristics of victims, harm methods, and causes behind suicide and self-harm.

Case	Age in Years	Education	Household Income (PKR)	Harm Method	Immediate Cause Behind the Attempt	Underlying Cause
**R9**	18	Higher Secondary	20 K	Came under a train	Family violence, psychologically tortured, and abused	Insult
**A2**	21	Middle	21 K	Black Rock	Mentally tortured and badly labeled by his brother’s wife	Insult
**H11**	20	Illiterate	10 K	Black Rock	Stepmother’s abuse and insult	Insult
**R12**	16	Illiterate	12 K	Insecticide	Husband insulted (marital stress), psychologically tortured	Insult
**T4**	25	Illiterate	15 K	Organophosphorus (pesticides)	Husband insults in front of family members	Insult
**T10**	19	Secondary	25 K	Hanged	Boyfriend cheats on her	Love Marriage Failure infidelity
**S13**	24	Secondary	14 K	Black Rock	Escape with boyfriend, destroy parents’ reputation (honor)	Love Marriage Failure
**N1**	20	Secondary	25 K	Insecticide	Spouse’s family disagreed about love marriage	Love Marriage Failure
**U7**	20	Illiterate	50 K	Hanged	Violence and boyfriend’s death	Love Marriage Failure
**K3**	20	Primary	13 K	Black rock	Exchange marriage does not satisfy desire	Forced Marriage
**E6**	19	Middle	15 K	Nail polish remover	Does not agree to marry an old man	Forced Marriage
B6	18	Higher Secondary	20 K	Firearm	Marriage	Arranged Marriage
**A7**	21	Middle	21 K	Hanging	Marriage without consent	Forced Marriage

## Data Availability

The original contributions presented in this study are included in the article. Further inquiries can be directed to the corresponding author(s).
